# Emotional Regulation in Mothers and Fathers and Relations to Aggression in Hong Kong Preschool Children

**DOI:** 10.1007/s10578-021-01165-y

**Published:** 2021-04-12

**Authors:** Eva Yi Hung Lau, Kate Williams

**Affiliations:** 1 Department of Early Childhood Education, The Education University of Hong Kong, Hong Kong, Hong Kong SAR; 2 School of Early Childhood and Inclusive Education, Queensland University of Technology, Brisbane, Australia

**Keywords:** Reappraisal, Suppression, Aggression, Child gender, Hong Kong Chinese

## Abstract

This study explored the associations among emotional regulation in mothers and fathers and preschool children’s physical and relational aggression using a Hong Kong Chinese sample. This study also explored whether child gender would moderate the association between parental emotional regulation strategies and children’s physical and relational aggression. Participants were 168 children aged 4–6 years. Parents reported on their own emotional regulation approaches and kindergarten class teachers rated children’s aggression 6 months later. Path analyses showed that higher levels of reappraisal and lower levels of suppression by mothers was associated with higher levels of child relational aggression. There were no significant associations among fathers’ emotional regulation and children’s aggression. Results from multi-group analysis showed that there were no significant moderation of the associations by child gender. Results highlight the importance of mothers’ emotional regulation in child aggression and suggest that the maladaptive consequences of emotional suppression are culturally relative.

## Introduction

Aggression, defined as behavior intended to harm others, is one of the most common types of childhood behavioral problems [1, 2]. Physical and relational aggression are the two most common forms of aggression used by children during the early childhood years [3]. Whereas physical aggression involves harming or threatening harm by means of physical damage (e.g., hitting and pushing), relational aggression is defined as harm or the threat of damage that is intended to manipulate or damage peer relationships (e.g. spreading rumors and threats to withdraw friendship) [4]. Aggressive children have been found to have high levels of psychological distress, low levels of prosocial behavior, and high levels of peer rejection [5]. Because of the numerous difficulties experienced by aggressive children, antecedents of childhood aggression are crucial to understand for developing prevention and intervention programs.

Parenting and family experiences are a key context in which children’s socio-emotional behaviors develop [6–9]. The parenting environment is especially influential during the preschool years when children are most open to parental influence [10]. In particular, studies have identified parental emotional regulation approaches as an important influencing factor for child behaviors and developmental outcomes [11–13]. Although there has been an increasing number of studies in the area of mothers’ emotional regulation and children’s externalizing behaviours [14–16], they are limited in their cross-sectional design, the lack of examination of fathers’ influence, and lack of representation of non-Western samples. To the best of our knowledge, no studies have investigated the precise role of parental emotional regulation in the development of children’s physical and relational aggression. This study explores associations of mothers’ and fathers’ emotional regulation and the use of physical and relational aggression by young children using a Chinese sample. This study will inform the understanding of how parental emotional regulation among Chinese parents affects child’s physical and relational aggression for developing practices to prevent or intervene children’s aggression through parental emotional regulation.

Emotional regulation refers to the processes responsible for monitoring, evaluating, and modifying emotional reactions to accomplish one’s goals [17]. These skills are considered a cornerstone of psychosocial functioning, with poorer emotional regulation implicated in many health and mental disorders [18, 19]. Two specific emotional regulation strategies in adults are commonly conceptualised and measured. Cognitive reappraisal refers to the ability to change thoughts associated with an emotion-inducing event in such a way as to modify its emotional impact [20]. Reappraisal is generally considered a highly adaptive emotional regulation approach associated with more positive psychosocial outcomes [21]. Emotional suppression refers to a strategy whereby individuals refrain from acknowledging or expressing emotions in an effort to regulate them [20] and is typically considered a maladaptive approach associated with poorer psychosocial outcomes over time [21].

Although parental emotional regulation is suggested to be important in shaping how parents act as emotion socialization agents for their children, it remains an understudied topic [22, 23]. Numerous studies have demonstrated the links between parents’ self-regulation and children’s outcomes [24–26]. However, only a few studies [14–16] have examined the links between parental use of emotional regulation strategies and children’s social behaviours, such as externalizing (e.g., impulsivity and hyperactivity) and prosocial (e.g., helping and sharing) behaviour, with none addressing childhood aggression specifically. In general, these studies find that higher use of adaptive emotional regulation approaches, including reappraisal, and lower use of maladaptive emotional regulation strategies, including suppression, by parents is associated with more positive social-emotional development in children. For instance, Crespo and colleagues [14] studied 454 American mothers and their children aged 3–7-years-old. These researchers found that children’s difficulties with emotional regulation mediated the relation between mothers’ lower emotion awareness and both internalizing and externalizing, whereas there was no significant direct associations between maternal difficulties with emotional regulation and children’s externalising behaviour problems, Specifically, maternal difficulties with emotional regulation were associated with higher levels of emotional negativity in children which in turn was associated with more externalizing behaviour problems in children. In an intervention study with 139 Australian children aged 29–83 months and their caregivers (93% mothers) referred for parenting support, it was found that increased use of cognitive reappraisal by caregivers was associated with a decline in children’s externalizing behavior problems [16]. A further recent study documented that higher levels of emotional suppression in caregivers (86% mothers) was associated with fewer prosocial behaviours in 3-year-old children in the US, with reappraisal by caregivers having no significant association [15].

In relation to childhood aggression specifically, parental adaptive emotional regulation, characterised by high levels of reappraisal and low levels of suppression, might reduce a child’s use of aggression through modelling processes and an influence on parenting behaviors. First, children may observe and imitate their parents’ emotional regulation capacities [27, 28]. It is possible that parents’ own emotional profiles and interactions implicitly teach children which emotions are acceptable and expected in the family environment, and how to manage the experience of those emotions [11]. It is also possible that parental emotional regulation creates a family emotional climate through which children will learn the appropriateness of emotional displays [29, 30]. As a result, parents’ modelling of poor emotional regulation approaches and children’s social references of these regulation strategies may contribute to children’s poor regulation. In fact, children with behavior problems have consistently been found to have limited emotion understanding, to display more negative emotions, and to have poorer capacity to regulate these emotions [31, 32].

Second, parents’ emotional regulation will affect their parenting practices. Parenting often involves numerous challenges and demands that require parents to remain calm and maintain positive affect in order to set clear limits for child behaviour and consistently follow through on stated rules. For parents with adaptive emotional regulation, they may be more responsive to their children’s emotional needs and emphasize empathic goals and problem solving strategies in the processes of socialization, which nurture their children to regulate negative emotions and undesirable tendencies that may result in aggression [33–35]. On the other hand, parents with poorer emotional regulation capacities may show higher levels of hostility and rejection and lower levels of emotional availability, which creates a context that is less supportive of children’s emotional and behavioural adjustment [24, 26, 36, 37].

In addition to a notable lack of research on the role of parental emotional regulation strategy use and children’s relational and physical aggression overall, there is a distinct lack of examination of mothers vs fathers and moderation of any effects by child gender. Given the complexity of family dynamics, delineating effects by parent and child gender is crucial in child and family studies. Traditionally, Chinese fathers have been regarded as the strict parent who is responsible for disciplining children in the family, with their general involvement in other aspects of parenting considered lower than mothers’ [38]. However, because of modernization and westernization, families in China, including Hong Kong, have undergone numerous changes in more recent years. For instance, gendered parenting roles have become less pervasive as most Hong Kong women aspire to obtain higher education and achieve an active working life [39, 40]. As a result, Chinese fathers are becoming more involved in child rearing and are considered important socializing agents in children’s development [40–42].

The role of fathers’ emotional regulation strategies in children’s social emotional development, particularly in aggression, has been largely unaddressed in the research literature to date. Contemporary fathers, including Chinese fathers, aspire to be actively involved in their children’s development [42]. Studies have also documented the positive influence of fathering on child developmental outcomes [43] and have shown a significant relation between fathers’ ER and their parenting practices [44]. Hence, understanding how fathers’ parenting can be promoted from infancy by facilitating prenatal ER is considered critical.

Available evidence from work examining fathers’ parenting and self-regulatory approaches more generally yields mixed findings. Some studies suggest that fathers’ behaviors exert a stronger influence than mothers’. For example, compared to mother-infant interactions, father-infant interactions have had stronger associations with infants’ [45] (Portugal) and toddlers’ emotional regulation [46] (United States); and paternal, but not maternal psychological aggression predicted children’s emotional regulation in middle childhood [47] (China). Still other studies suggest that fathers exert an equal influence to mothers. For example, fathers’ emotional dysregulation was found to be as likely to influence levels of parent–child aggression in families with pre-schoolers in the United States as mothers’ dysregulation [48]. It is clear that more research is needed to better establish the role of fathers’ emotional regulation.

Child gender is also an important consideration and may moderate relations among parenting and children’s social-emotional development. Previous studies have suggested that boys and girls differed in their response to maternal emotional expression [49, 50]. Some literature suggests that parents are more likely to socialize daughters’ emotional and social development than sons, and that girls are more sensitive to family affective environment than boys [11, 51]. However, other research has documented that both mothers and fathers used more emotional language with boys than with girls [52], and lower levels of maternal sensitivity have been found to be particularly detrimental to boys in terms of their development of externalizing behavior problems [53]. It is clear that further research on the role of child gender in the context of emotional regulation by both mothers and fathers is warranted.

Differences in cultural and parenting norms are likely to influence parents’ emotional regulation approaches and the ways in which they influence children’s social-emotional development. While most research to date on parents’ emotional regulation and children’s social-emotional functioning has been done with Western participants, there is a growing field of research with Chinese participants and other collectivist cultures. However, these studies have yielded mixed results. On one hand, it is possible that although suppression has typically been found to be maladaptive, the maladaptive consequences of suppression may be culturally relative. Specifically, among collectivist cultures, freely expressed emotions, particularly negative emotions, are often discouraged as they are thought to disrupt group cohesiveness and social harmony [54]. For example, Butler, Lee and Gross [55] and Cheung and Park [56] found that suppressers who held Western-European values had poorer social and emotional outcomes during interpersonal interactions than did suppressers who held Asian values. Similarly, Yuan et al. [57] and Soto et al. [58] found that suppression may be more adaptive among participants in the Chinese context than in Western cultures. In contrast, other studies have identified the costs associated with suppressing with Chinese and Chinese American samples, including psychological distress, social functioning difficulties, and health issues [59, 60]. Given the limited existing evidence base and the to-date mixed findings, the present study is necessary to better understand the associations between parental emotional regulation and child aggression within the Chinese context.

Researchers have increasingly realized the importance of the fathers’ role in children’s development [61–63]. However, relatively less studies have examined how fathers’ emotional regulation strategies may be associated with children’s aggression and even fewer studies have been conducted in a Chinese context. The present study used a Hong Kong Chinese sample to explore the direct relations between both mothers’ and fathers’ emotional regulation and child aggression. This study also explored whether the association between mothers’ and fathers’ emotional regulation and child aggression would be moderated by child gender. Based on previous work, it was hypothesized that higher use of reappraisal by both mothers and fathers would be related to lower physical and relational aggression in children. Because of the cultural context, we hypothesized that higher levels of suppression used by mothers and fathers would also be related to lower physical and relational aggression in children, in our study conducted in a collectivist culture. Given the mixed findings to date about the role of fathers and the effect of child gender, we had no specific hypotheses about the nature and strength of father associations compared to mother associations on boys versus girls.

## Method

### Participants and Procedure

In the present study, the participants were recruited using a stratified and convenience sampling strategy. Specifically, the number of classes invited in each of the three main territories, namely Hong Kong Island, Kowloon, and New Territories, varied so that the percentages of participants from each territory resembled the percentages of the Hong Kong population in each area. In 2016, approximately 17.1, 30.6 and 52.3% of the Hong Kong population lived in Hong Kong Island, Kowloon, and New Territories, respectively [64]. All kindergartens in Hong Kong are privately run and provide services for children from three to six years old. Nearly all children in Hong Kong start kindergarten at age three and they are grouped into classes according to their age (e.g., 3–4-year-old, 4–5-year-old, and 5–6-year-old). Invitation letters were sent to kindergartens and phone calls were made to the principals. Five kindergartens (two from each of Hong Kong Island and New Territories and one from Kowloon) agreed to participate in the study. A total of 17 classes from the 5 kindergartens were invited to participate. All teachers (N = 17; 16 females) of the participating classes consented to participation.

The teachers distributed introductory letters about the study to all parents in their class. A total of 175 parents provided consent to participate in this study (Hong Kong Island: 18.3%, Kowloon: 34.3%, and New Territories: 47.4%). Unfortunately, the consent rate was not available because some of the kindergartens failed to provide information about their class sizes. Seven of the children were excluded due to their special educational need (e.g. social functioning and cognitive functioning). The final sample included a total of 168 children from five 4–5-year-old classes (*N* = 35) and 12 5–6-year-old classes (*N* = 133). The children (52% girls) were on average 61 months old (*SD* = 5.51 months).

The mothers and fathers of the children were on average 35.6 years (*SD* = 4.93) and 39.9 years (*SD* = 6.12) old respectively. The median education level that the participating parents had attained was secondary education (58.9% of mothers and 55.4% of fathers), which was also the median education level of the Hong Kong population in 2016 [64]. In terms of marital status, 149 sets of parents reported themselves as married, five sets of parents as divorced, one set of parents remarried and six sets of parents selected “others” as their marital status (i.e. single or separating). The median monthly household income of the participating families was HK$27,000 (*M* = 29,450; *SD* = 18,713; US$1 = HK$7.78), which was slightly higher than the median monthly household income of Hong Kong families (HK$24,890) [64].

In this study, data were collected at two time points with a six-month interval in order to examine the predictive effect of parent emotion regulation (Time 1 predictor) on subsequent child aggression (Time 2 outcome) between individuals. The six-month interval in between Time 1 and Time 2 data collection would ensure sufficient time for the child behaviors to develop within the project timeframe for testing the hypothesized relations. At Time 1, approximately 3 months after the school year has started, we invited parents to complete questionnaires related to their family background and their own emotional regulation. Among the 168 families, 151 (90%) of them returned both father and mother questionnaires, seven families only returned the mother questionnaire, three families only returned the father questionnaire, and seven families failed to return any parent questionnaires. At Time 2, approximately 6 months after the first time point, the lead teacher in each participating class was invited to provide ratings of physical and relational aggression for each participating child, except one child who changed school after Time 1. Completed questionnaires (*N* = 167) were successfully obtained from all participating teachers. Each family received a supermarket coupon at the value of USD6 as a token of appreciation for their participation.

### Measures

#### Parental Emotional Regulation

Parents’ emotional regulation was assessed with the *Emotion Regulation Questionnaire* [65]. Each parent reported their own emotion regulatory processes using the 6-item *reappraisal* subscale (e.g., “I control my emotions by changing the way I think about the situation I am in”) and 4-item *suppression* subscale (e.g., “I keep my emotions to myself”) subscale. Each item was rated on 7-point scale (1 = *strongly disagree,* 7 = *strongly agree*). In the present study, the two subscales achieved adequate internal consistency for both mothers (reappraisal: 0.85; suppression: 0.74) and fathers (reappraisal: 0.88; suppression: 0.75). The scores for reappraisal and suppression were generated by averaging the items within each subscale. Higher scores indicated higher levels of reappraisal and suppression.

#### Child Aggression

The *Preschool Social Behavior Scale* [66] was used to assess teachers’ rating of children’s *physical aggression* (8 items; e.g., “This child pushes or shoves other children”) and *relational aggression* (9 items; e.g., “This child tells others not to play with or be a peer’s friend”) on a five-point Likert scale (1 = *never*; 5 = *always*). Similar to previous studies involving Chinese populations (e.g., [42]), the two subscales showed strong internal consistency (physical aggression: 0.92, and relational aggression: 0.94) in this study. The scores for physical aggression and relational aggression were generated by averaging the items within each subscale. Higher scores indicated higher levels of aggression.

### Approach to Analysis

All analyses were conducted in Mplus Version 7.11 [67]. We first tested a whole sample model followed by a multi-group model that produced separate path estimates for boys and girls and tested sequentially constrained models to compare model fit using BIC. Missing data was minimal ranging from zero missing data on child aggression variables to 8.3% missing data for fathers’ emotional regulation. Missing data was handled through use of maximum likelihood estimation within the models.

It is important to note that outcome data for teacher-reported children’s aggression is nested within classrooms. Intra class correlations for teacher reported aggression were 0.26 and 0.31 for physical and relational aggression respectively, creating corresponding variance inflation factors of 3.27 and 3.73. However, due to sample size, the model is underpowered to account for clustering in estimation. This increases the chance of Type 1 error and is acknowledged as a limitation of the study. Results should therefore be interpreted with caution and considered exploratory until results are produced by replication studies with larger sample sizes or non-clustered outcome data.

## Results

### Descriptive Statistics

Bivariate correlations among all variables are provided in Table 1. Children’s physical and relational aggression as reported by teachers was strongly correlated. Boys displayed more physical aggression but gender was not correlated with relational aggression. Being a mother of a boy and a more highly educated mother was associated with less use of suppression. Mother’s use of reappraisal showed small but significant correlations with mothers’ use of suppression and fathers’ use of reappraisal. Father’s reappraisal and suppression emotional regulation were also moderately correlated.

**Table 1:** Bivariate correlations among all variables modelled

		1	2	3	4	5	6	7	8	9
1	Boy	1								
2	Age (months)	− .06	1							
3	Maternal education	.09	.06	1						
4	Mothers’ reappraisal	.08	− .04	.07	1					
5	Mothers’ suppression	− .16*	.05	− .17*	.21*	1				
6	Fathers’ reappraisal	− .02	.04	.02	.20*	− .05	1			
7	Fathers’ suppression	− .06	− .03	− .11	− .02	.03	.41**	1		
8	Child physical aggression	.25*	.10	− .04	.08	− .06	− .06	− .01	1	
9	Child relational aggression	− .03	.08	− .04	.11	− .12	− .01	.01	.74**	1
	Range		46–71	1–6	2–7	1–6.33	1–7	1–6.75	1–3.38	1–3.89
	Mean		60.97	2.63	5.03	3.82	4.93	4.19	1.29	1.62
	*SD*		5.51	.92	.92	1.07	1.03	1.09	.51	.71
	*α*				.85	.74	.88	.75	.92	.94

*p < .05; **p < .01

### Mothers’ and Fathers’ Emotional Regulation and Children’s Physical and Relational Aggression

**Fig. 1:**
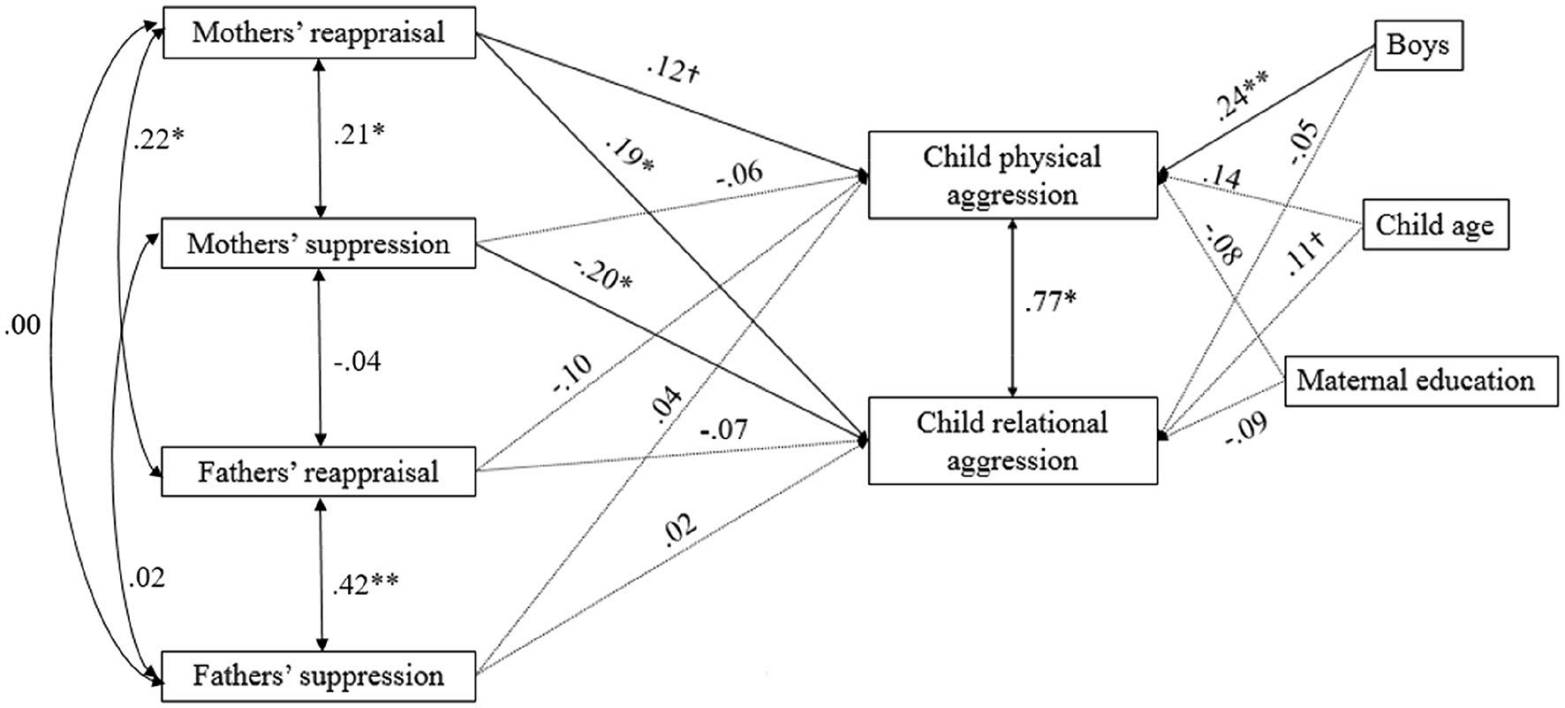
Path model for the effect of early mothers’ and fathers’ self-reported emotional regulation strategies on later physical and relational aggression in children, as reported by teachers. Fit indices: *x*^2^ = 16.81, *df* = 12, *p* = .16; RMSEA = .05, CI = .00 to .10; CFI = .97, BIC = 2297.683. Dotted lines indicate non-significant path estimates. ^†^*p* < .10; *p < .05; **p < .01. All estimates are standardized

The path analysis for the associations among mothers’ and father’s self-reported emotional regulation approaches and children’s teacher-reported aggression, was a good fit to the data (Fig. 1). Higher levels of reappraisal and lower levels of suppression by mothers was associated with higher levels of relational aggression in children. There was a marginal association with higher levels of mothers’ reappraisal associated with higher levels of children’s physical aggression. There were no associations between fathers’ emotional regulation strategies and children’s aggression. Because this model controlled for mothers’ emotional regulation, we also tested a model that included *only* fathers’ self-regulation in relation to children’s aggression. No significant associations among fathers’ emotional regulation and children’s aggression were found in this independent model.

### Child Gender as a Moderator

**Fig. 2:**
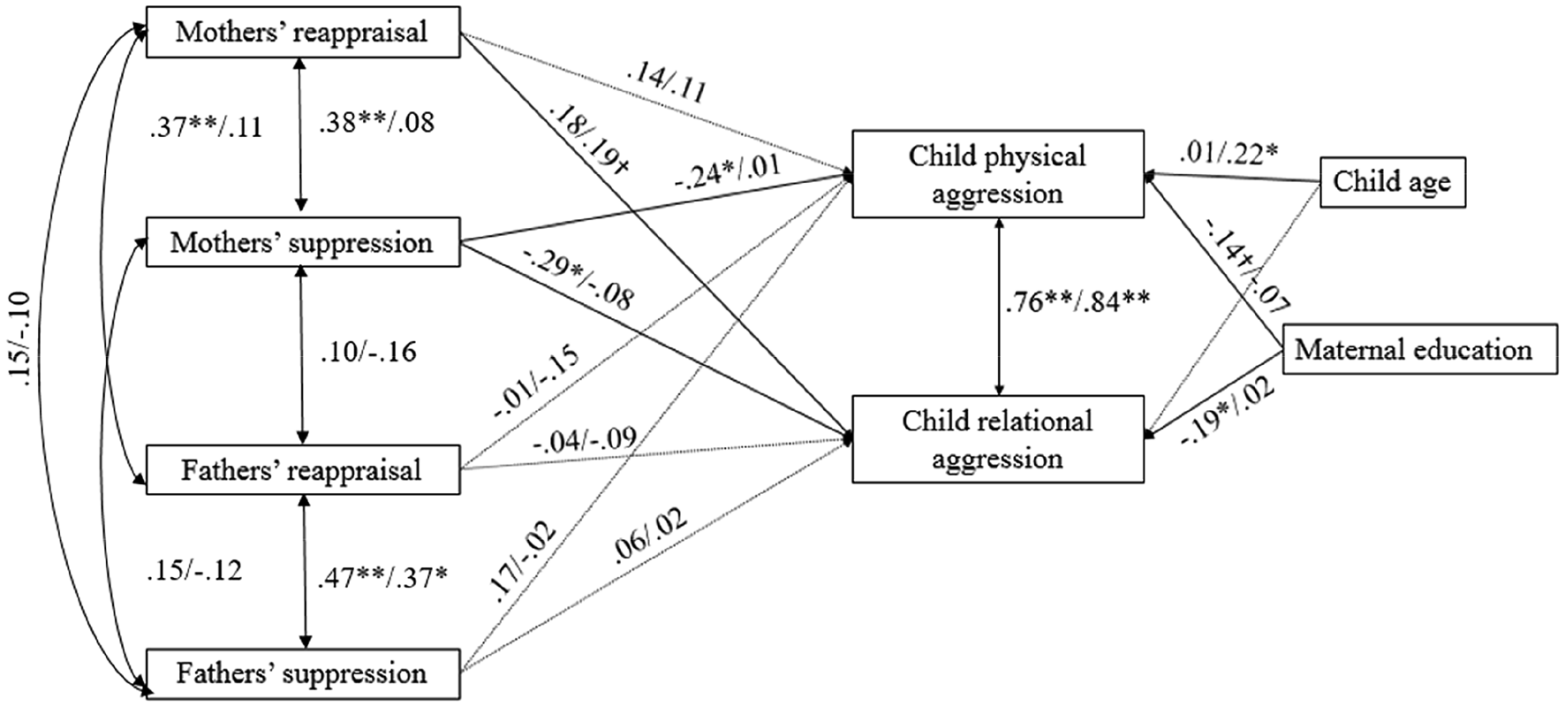
Path model for the effect of early mothers’ and fathers’ self-reported emotional regulation strategies on later physical and relational aggression in children, as reported by teachers. Fit indices: *x*^2^ = 10.86, *df* = 16, *p* = .81; RMSEA = .00, CI = .00 to .64; CFI = 1.00, BIC = 2359.30. ^†^*p* < .10; *p < .05; **p < .01. All estimates are standardized. Paired parameters indicated standardized estimates for girls/boys. Differences across gender are not significant

The multi-group analysis for boys and girls was also a good fit to the data (Fig. 2). However, constraining paths to be equal across gender did not worsen model fit as assessed by BIC, thus there is no evidence that there is statistically significant moderation of associations by gender. However, interpretation of the estimates yields some interesting points. In this model the associations between mothers’ reappraisal and higher child relational aggression, and mothers’ suppression and lower child relational aggression remained. The association at the whole group level between higher use of suppression by mothers and lower levels of child relational aggression appeared to be driven primarily by an influence on girls in particular. This influence of mothers on girls was also reflected in a new association that did not appear in the whole sample model, with mothers’ use of suppression being related to lower levels of physical aggression in children, particularly for girls.

## Discussion

Emotional regulation skills develop across the lifespan but may take on particular relevance during parenthood when parents must manage their own emotions while simultaneously facilitating their children’s regulation of emotions and behaviors [68]. The present study explores an important gap in the literature by examining the contribution of parental emotional regulation on children’s aggression using a Chinese Hong Kong sample. Consistent with existing studies [14–16], the present study found that parents’ emotional regulation is directly relevant to children’s social behaviors. More specifically, we found that maternal, but not paternal, emotional regulation was related to child aggression. Such findings are consistent with previous research suggesting that mothers have a stronger influence on child socioemotional outcomes because they are more likely to be involved in the development of children’s ability to cope or manage their emotional expression and experience [51, 69, 70]. A number of implications for future research and practice are discussed below.

Contrary to our hypothesis, maternal reappraisal was found to predict higher levels of physical and relational aggression. Cognitive reappraisal involves changing thoughts associated with an emotion-inducing event that has been acknowledged [20]. For example, a parent who is experiencing a stressful work event may use cognitive reappraisal to regulate the induced negative emotions by acknowledging and accepting her negative emotions, followed by an evaluation of the benefits or opportunities that the event may bring. These processes are important prerequisite to the execution of a solution. Compared to that of emotional suppression, the the internal nature of cognitive reappraisal is more complex and may not provide a context in which this regulatory approach could be learned [6]. Socialization involves parents’ direct and indirect influences on children’s experience as well as regulation of emotions and behaviours through parents’ own expression of emotion, responsiveness, and guidance [22, 71–73]. Through observing their mothers’ use of reappraisal in regulating their own emotions, children may learn to be aware and accept their own emotions indirectly, but not necessarily learn to manage their emotions through the execution of a solution effectively. As a result, a lack of direct guidance and instruction (e.g., emotion coaching) from the parent to the child in a healthy, age-appropriate way may leave children with fewer strategies for managing their negative emotion or solving peer conflicts, in which they may use more aggression to express the negative emotions induced by their interpersonal problems that they have identified and experienced. The above findings highlight the importance of providing trainings for parents and children so that they can both learn to use effective emotional regulation strategies directly. Clinicians and parent educators can also coach parents to teach children how to manage their emotions through the execution of a solution to resolve peer conflicts, in addition to only identifying and being aware of their negative emotions. As parents’ reappraisal processes may not be explicit and verbalised, parents should also be supported to verbalise the emotional regulation strategies they use so that they can influence children’s own emotional regulation and aggression.

Consistent with existing studies that the maladaptive consequences of suppression may be culturally relative and that suppression is not related to externalizing behaviors such as aggression in collectivist cultures [55–58], we found evidence supporting our hypothesis that higher levels of maternal suppression predicted lower relational aggression in children. As freely expressed emotion, particularly negative emotions, is often discouraged for maintaining group cohesiveness in the Chinese context [54], mothers may try to protect their children from experiencing adverse responses by hiding their feelings from their children. As a result, the use of suppression may be somewhat normalized and accepted by children in Hong Kong. Specifically, children may learn by observing and interacting with their mothers that suppressing their emotions and refraining from acting in ways that would disrupt social harmony is appropriate and hence, reduces children’s use of relational aggression. Although suppression was found to decrease children’s relational aggression as a negative expression, numerous studies have suggested the use of suppression as a risk factor for internalizing problems such as increased depressive and anxiety symptoms in both Western [74, 75]and Asian collectivist cultures [59]. It has also been suggested that suppression may hinder parents’ ability to respond sensitively and appropriately to children’s emotions, which can be particularly damaging in the context of parent–child relationships [76]. Therefore, more studies investigating parental suppression, child suppression, child aggression and child internalizing problems in the Chinese context are needed to better understand the nature and influences of suppression in diverse cultures. Nevertheless, the findings suggested that parents with low levels of suppression in the Chinese culture may be a risk factor for children’s aggressive behaviors, clinicians and parent educators should encourage a balance between appropriate parental emotional expression and suppression.

In this study, neither the model with both fathers and mothers included, or an independent model with only fathers, yielded significant associations between paternal emotional regulation strategies and children’s aggression. The finding is in contrast to more general studies on the role of fathers in children’s social-emotional development which suggest an at-least-equal-to, if not greater, influence as mothers [45–47]. However, most prior studies have been conducted with Western samples and none, to our knowledge, have specifically examined paternal emotional regulation and childhood aggression. It is likely that our findings reflect a context in which mothers are the main caretakers of children and thus more likely to be involved in the socialization of children’s emotional and social behaviors. As such, it is possible that the level of paternal involvement would moderate the relation between paternal emotional regulation and child aggression, in which fathers’ emotional regulation would only influence child aggression when they are highly involved in their development. Nevertheless, as role expectations for mothers and fathers continue to change in Chinese societies, further research is required to replicate these findings and further understand potential mediators of these associations. For example, while paternal emotional regulation was not a significant influence in the current study, it may have an important influence on children’s aggressive behaviors through externalized parenting acts by fathers including expressed warmth, hostility, and engagement in home learning activities. Importantly, evidence produced by the current study, along with others, can be used to inform the target audience and behaviors of parenting support programs that aim to influence child developmental outcomes. For example, differentiated interventions might be needed for mothers vs fathers, with different targets for knowledge and behavior change.

While there was no statistically significant evidence of moderation by child gender in this study, there was a trend toward stronger associations between maternal emotional regulation and girls’ aggression, which may have reached significance with a larger sample size. In particular, mothers’ use of suppression was associated with lower levels of both relational and physical aggression for girls. Girls may be particularly susceptible to the socialization of suppression as they use their mothers as role models to guide behavior. As discussed above, suppression in the collectivist culture may act to reduce overt aggression and maintain social cohesion, and this may be particularly so for girls. This finding reflects prior research that suggests girls are more sensitive to mothers’ emotional expression and parenting approaches than boys [50, 77]. Further research is needed to develop a more comprehensive understanding of a range of parental influences in relation to the development of childhood aggression. The implications of differential associations for boys and girls are that different intervention targets and approaches for boys and girls might be needed to intervene on or prevent aggressive behaviors.

## Limitations and Future Directions

This study provides initial evidence suggesting that parental use of emotional regulation strategies may be an important influence on child aggression. Our findings add to the growing body of literature indicating that aspects of parental self-regulation are related to important child outcomes and influence the context in which children are being raised. This study exhibited several notable strengths such as the collection of data at two time points and the inclusion of both mothers and fathers. Nevertheless, several limitations of this study must be acknowledged. First, this study was limited by the examination of the direct associations between parental emotional regulation strategies and forms of child aggression, without the inclusion of potential mediators such as the quantity of parental involvement and quality of parenting behaviors. In practice, it is likely that parental emotional regulation does not directly influence how aggressively children behave with peers, but instead influences this more distal outcome by altering parenting strategies and children’s socioemotional functioning [78]. In particular, the unexpected findings of the positive relations between maternal reappraisal and child physical and relational aggression could be better articulated by including variables such as parents’ emotion coaching strategies. Similarly, while paternal emotional regulation was not related to any child outcomes, the importance of the paternal emotion variables for children’s aggression might become more apparent if parenting behavior or children’s emotional regulation is included as the mediating mechanism. It is also possible that fathers’ emotional regulation would only have an impact on child aggression when they are highly involved. Hence, future research with a focus on assessing these potential mechanisms is needed to examine such complex, multiple mediated and moderated effects. Second, parental self-reports of emotional regulation may be different in a setting that induces strong affect as opposed to the general context in which it was assessed in the current study. Therefore, the conclusions that were drawn from this study regarding the processes by which parental emotional regulation contributes to child aggression should be interpreted with caution. Future studies should assess parental emotional regulation during parenting as it is the context in which children are likely to be affected by their parents’ emotional regulation strategy use. Third, as in previous studies in both Western [79] and non-Western [80] contexts, our study involved a sample of pre-schoolers who displayed relatively low levels of aggression. The results may be different when examining children with more elevated aggression scores. Relatedly, we only used teacher reports for assessing child aggression. Future studies should consider replicating this study within a more clinical sample and using a multi-informant approach or utilize observation methods to obtain data of child aggression. Fourth, our ability to detect potentially small but important effects was limited by our relatively small sample size. Fifth, with children nested within schools and within classrooms, the possible non-independence of teacher ratings of child aggression should be noted. Hence, results should be considered exploratory and interpreted with caution. Future studies should consider replicating this study by employing larger samples or non-clustered outcome data to examine similar questions. Sixth, finally, as Hong Kong is considered the most westernized city in China, our findings may not be generalized to parents in Mainland China. Future studies should be conducted in other areas in Mainland China before the findings are generalized.

## Summary

This study is the first, to our knowledge, to document the associations between maternal and paternal emotional regulation strategy use, and children’s relational and physical aggression, in a Chinese sample. The findings document important associations between maternal emotional reappraisal and suppression and children’s aggressive behaviors. The study makes an important contribution to the limited evidence base in this field, and suggests that further research is warranted to better understand the specific familial mechanisms involved in the development of childhood aggression. As knowledge grows in this area, more targeted and effective prevention and intervention strategies that aim to reduce aggressive behaviors in childhood can be developed.
